# Formulation and Optimization of Complementary Food Based on Its Nutritional and Antinutritional Analysis

**DOI:** 10.1155/2022/1126031

**Published:** 2022-10-17

**Authors:** Elsabet Getachew Aynalem, Ramesh Duraisamy

**Affiliations:** Department of Chemistry (Industrial Chemistry Division: Food and Sugar Technology), College of Natural and Computational Sciences, Arba Minch University, Arba Minch, Ethiopia

## Abstract

This study focuses on formulating and optimizing complementary foods using oat, yellow maize, chickpea, avocado pulp powder, and sugarcane Jaggery to obtain nutritious infant food. Different proportions of the composite food were optimized using Minitab v.19 software upon mixture design by keeping the constant amount of Jaggery (10%). The formulated composite foods had a better proximate composition, minerals, vitamins, and lower amounts of antinutrients. Most attributes have shown significant differences (*p* < 0.05) among those formulations. The better-optimized formulation is selected from nutritional and antinutritional values through overlaid contour design. The study concludes the optimal complementary food composition ratio: oat (40 g), chickpea (25.81 g), yellow maize (13.78 g), avocado powder (10.4 g), and Jaggery (10 g). Thus, the proposed formulated product becomes nutritious complementary food that may help children's and medium-age hold communities.

## 1. Introduction

The World Health Organization (WHO) describes complementary feeding as a process starting when breast milk alone is no longer sufficient to meet the nutritional requirements of infants. Therefore, other foods and liquids are needed, along with breast milk [1]. There is a requirement by which complementary foods should include high energy density, balanced protein composition (containing all essential amino acids), and micronutrients like vitamins and minerals (iron, folic acid, and calcium) [2]. Cereals and grains are common foods in most human diets in developed and developing countries, providing the main proportion of dietary energy and nutrients. It contains 75% carbohydrates, primarily starches, and about 6-15% protein, contributing to more than 50% of the energy supply [3, 4].

In Ethiopia, homemade baby foods are prepared primarily from cereal grains, starting around six months. It is ready in the form of solid and liquid (porridge and gruel). These complementary foods typically lack essential amino acids (protein) and micronutrients, which lead to different nutrient deficiency diseases. They are high in antinutrients, which affect the bioavailability of essential minerals. Furthermore, these cereal-based complementary foods have high fiber and less nutrient density, which immediately fill infants' stomachs since high-fiber foods are bulky and low in caloric density [5].

Avocado is a super nutritious fruit that contains essential nutrients for infants but is highly susceptible to microbial spoilage and stored for a short period. Due to this, it needs a proper value addition process like chemical treatment and drying at a lower temperature. Such studies are crucial to coming up with tangible means like blending and compiling with various foodstuffs to produce nutritional rich and good sensory value food products that can promote appetite and habit of consumption. Sugarcane contributes to 90% of the world's sweetener production. Although sugar is added in processing such foods, there is minimal literature on the case of using Jaggery as a sweetener in complementary foods [6]. Worldwide, More than 70% of Jaggery produced from India by processed the sugarcane [4]. Most complementary foods in the market are costly and cannot be economically viable for low-income families [7]. Thus, this research intended to formulate nutritious food products using harmless sweeteners and value-added food products from readily available cereal grains, legumes, fruit, and Jaggery to help overcome nutrient deficiency problems by filling the nutrient gap required by infants. Furthermore, using Jaggery instead of table sugar in the proper amount helps to obtain essential nutrients in addition to its sweetness.

## 2. Materials and Methods

### 2.1. Raw Material Selection

Raw materials were selected based on Codex Alimentarius guidelines [8]. Accordingly, the blend was composed of cereal and fruit-based ingredients widely used for preparing complementary food. Ingredients are used in this study based on their nutrients as follows: oat (carbohydrates-rich), chickpea (protein-rich), yellow maize (vitamin-rich), and avocado (fat-rich). Additionally, Jaggery was used as a sweetener in addition to supplementing micronutrients and improving the sensory qualities of composite blends. The components were packed in air-tight polythene bags and stored in a laboratory at room temperature until needed for processing.

### 2.2. Raw Material Preparation

#### 2.2.1. Oat Processing

The oats were cleaned using air aspiration. It was dried in an oven at 40°C, then dehulled and heated at 60°C five hours to develop flavor and deactivate microorganisms. Finally, grinding and sieving were performed using 250 *μ*m to get fine particles [9].

#### 2.2.2. Yellow Maize Processing

Yellow maize was winnowed, sorted, washed with tap water (3 : 1 water to sample ratio), and soaked for 24 hours at room temperature 25°C. It was removed from the water, washed thrice with water, and dried in an oven maintained at 60°C until <2% free from moisture [10].

#### 2.2.3. Chickpea Processing

It was hand-sorted and soaked (3 : 1 water to sample ratio) for 24 hours at room temperature (25°C). It was removed from the water, washed thrice with water, and dried in an oven maintained at 60°C until it was free from moisture (<2%). Dehulling and air aspiration was undertaken for removing the chickpea outer-layer shield. followed by grinding the pretreated chickpea using mortar and pestle [11].

#### 2.2.4. Avocado Powder Processing

Pure, fully ripe, and undamaged avocado fruit samples were selected and washed with pure water by applying appropriate soap. It was peeled and sliced using a stainless steel knife. Blanching was performed (under soaking) at 80°C with lemon juice (with 35% citric acid, which was used as an antibrowning agent) for 4 minutes, drained, and placed on aluminum trays. It was dried using an oven dryer at 45°C for 24 hours and packed using moisture-free polyethylene bag-appropriate packing material [12].

#### 2.2.5. Jaggery Processing

Sugarcane Jaggery was prepared using a three-step process: juice extraction, clarification, and concentration. Fresh sugarcane was collected from cultivation land in Mirab-Abaya, which is near the study area (Arba Minch town). The samples were washed with pure water and peeled using a knife, ground using a juice extractor, and extracted the juice. Filtration was followed by waiting (five minutes) to remove heavy impurities and fine bagasse after sedimentation. Clarification was started during the boiling stage at 75°C (to make hot liming) by adding lime (10% calcium oxide) and filtered again after cooling to remove impurities in the form of scum. The clarified juice was heated on a hot plate to raise the temperature with continuous stirring and monitored using a thermometer [13]. Through constant boiling (as with increasing the temperature gradually from 100 to 118°C), the brownish foam was observed at the top and disappeared with uniform boiling at >110°C, which gave a golden color. When the temperature reaches 105°C, the juice starts frothing. Then, the heat is carefully regulated to prevent caramelization by employing a thermostat. The syrup was constantly stirred to avoid charring and to drop over the pan's side. The concentrated juice was removed from the thermostatic hot plate, poured into aluminum foil, and molded with desired shape [14].

### 2.3. Formulation of Complementary Foods Using Experimental Design

Different mixing ratios prepared the formulation of complementary foods through the Minitab version 19 mixture design model. A mixture design is applicable when the response depends on the component ranges of the mixture; 17 different compositions (shown in Table 1) helped to select the best optimum compositions through other food quality parameters. The preliminary study was conducted, and based on those results, the constraints used are 40-44 g/100 g oat, 15-33 g/100 g chickpea, 12-23 g/100 g yellow maize, and 5-11 g/100 g avocado powder of upper and lower constraints with a constant amount of Jaggery (10 g/100 g) with the total amount of formulation as 100 g.

### 2.4. Nutrient Analysis of Raw Materials and Complementary Foods

The proximate compositional analysis, including percentages of fat, fiber, protein, carbohydrate, moisture, ash, and energy, was determined by [15] methods. Graphite furnace atomic absorption spectroscopy was used to estimate the mineral concentrations in the food samples. The *β*-carotene content was determined using a UV/Vis spectrophotometer by measuring the absorbance of the extract and the blank solution at 450 nm. Using the Beers Lambert law from measured absorbance data, its concentration was calculated in mg·l^−1^ [16].

Total phenol was determined by extracting the phenolic compound from the sample using methanol [17]. Total phenolic contents were expressed as gallic acid equivalents (GAE) in mg/100 g GAE (at 765 nm) using a UV/Vis spectrophotometer.

Vitamin C was determined by titration by preparing a standard vitamin C solution and using standard iodine solution as a titrant by adding starch indicator until the observation of blue color [18]. In all cases, sample preparation was processed based on a method designated by the AOAC method [15].

### 2.5. Analysis of Antinutrients of Formulated Complementary Food

Phytate content was determined as described by [19]. Follwed by extraction, filtration, titrated the sample solution with standard iron (III) chloride solution using ammonium thiocynate as an indicator. Then, end point (persistence of golden color for 5 minutes) was noticed. Phytate content was calculated using equation (1):

(1) $$\begin{array}{c} \text{Phytic acid}=\text{Titer value}\times \left(0.00195\right)\times 1.19\times 100, \end{array}$$

where 0.00195 is the weight of FeCl_3_.

Tannin was determined spectrophotometrically at 500 nm as described by [20]. Using the tannic acid (Merck chemicals, India, with 90% purity) solution standard, the calibration curve was used to determine the concentration [21].

Oxalate was determined by using the titration method using KMnO_4_ as a titrant after extraction [15]. Titration was performed until observation of blue color and persisted for at least one minute [22] and calculated as

(2) $$\begin{array}{c} \%\text{Oxalate}=\left(\frac{\text{Titer value}}{W}\right)\times 0.06303\times 100. \end{array}$$

The relationship, that is, 1 mL of KMnO_4_ solution = 0.006303 g oxalate, was used, where titer is the volume of KMnO_4_ consumed and *W* is the weight of the sample.

### 2.6. Statistical Analysis

The results of experimental designs of the proportions of models described by each response were validated, and the analysis of variance (ANOVA) helps to evaluate each response, the effect of each factor, and interactions among factors.

## 3. Results and Discussion

### 3.1. Optimization of Composite Complementary Foods by Its Proximate Composition

The proximate compositions of composite food samples' such as moisture, ash, protein, fat, fiber, carbohydrate contents, and gross energy were analyzed. ANOVA has been used, and characteristics and quantities evaluated the variants. Amongst, *p*-values and predicted *R*^2^ values are used and analyzed the responses. Generally, if the *p* value is less than alpha (<0.05) which is more significant, then the model of the parameter or interaction can be considered statistically significant. Tables 2 and 3 summarize the ANOVA *p* values of all the quantities of responses, such as proximate composition, antinutrients, vitamins, and minerals. The full cubic with special cubic models was used for the statistical data analysis of responses. These results were obtained from mixture components as model terms, and the mixture regression design model was selected as a model-fitting method [23].

Table 2 reveals that the changes in proximate compositional of samples to moisture, protein, fat, and carbohydrate (CHO) contents were found to be significantly influenced (*p* < 0.05) by the blending of the constituents. It shows a statistically significant (*p* < 0.05) relationship between the oat, chickpea, maize, and avocado pulp powder blending ratios. The other interactions had also shown significant changes. Still, 3-way interaction between oats, maize, and avocado powder has the least nonsignificant, which is proved by their *p* values (0.987 in fiber and 0.264 in energy) in this case. But, the overall selected model was found to a significant (*p* < 0.05), with the adjusted *R*^2^ values that ranged from 0.875 to 0.998 (shown in Table 2).

The cubic regression ANOVA results (Table 3) for this case also reveal that in copper and manganese, all interactions have not shown statistically significant difference (*p* > 0.05). It is confirmed with a lower *R*^2^ adjusted value, but iron, calcium, zinc, and magnesium in all cases of interactions show a significant difference (*p* value < 0.05) with *R*^2^ adjusted value from 0.977 to 0.992.

Table 4 describes that the results of phytate, oxalate, and *β*-carotene fit very well (*R*^2^ − adjusted > 0.95). It is confirmed that it provides a reasonably good explanation of the relationship between the independent factors (food components) and the responses of the processing of blending themselves. *R*^2^ values of polyphenol, tannin, and vitamin C were found to be 0.905, 0.890, and 0.893, respectively. Similarly, the cubic models for vitamin C, polyphenol, and tannin also fit the data well because the adjusted *R*^2^ is greater than 0.80. On the other hand, for polyphenol and vitamin C content, except for linear and quadratic models, all three interactions (cubic model) do not fit the data well (*p* > 0.05).

### 3.2. Optimal Responses Based on Contour Plate

**Figure 1:**
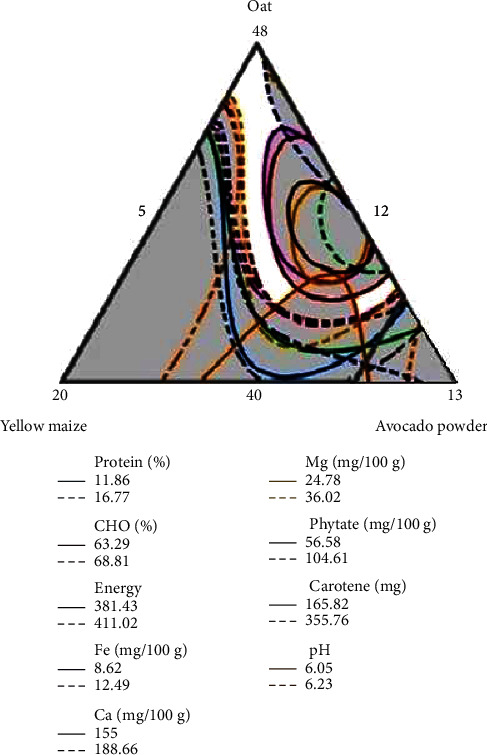
Overlaid contour plots that show the sweet spot. Notes: the white area shows the “sweet spot” that optimizes the response variables listed in the respective legends.

In the contour plot (Figure 1), the white “sweet spot” that optimizes the responses was determined using the researchers' lower and upper goals for a response [23]. For optimization, resolved responses that show significant differences in the blends were considered. The overlaid contour plot design (Figure 1) for proximate, antinutrient, vitamins, and minerals was obtained by considering significant values (*p* < 0.05) to find the optimization region using the contour plot design.

The overlaid contour plot was drawn using the protein, CHO, energy, Fe, Ca, Mg, phytate, and pH. All these responses were used and found the optimal region of food composnents of the current study.

### 3.3. Response Optimization Using D-Optimizer

A constrained D-optimal mixture design was used and predicted the best six optimum blending ratios of the food mixture components (mixture ratio proposed ranged in all optimum mixture: oat: 40-49 g/100 g, chickpea: 15-31.77 g/100 g, maize: 12.17-21.6 g/100 g), and avocado powder: 5-11 g/100 g with appropriate responses of each food quality parameters, which is presented in Table 5.

Different parameters were analyzed for the best-optimized product to compare the predicted and actual values. The obtained experimental result was parallel with the predicted value. From the proximate compositional analysis, protein, CHO, and fat contents were analyzed and received 15.12 g, 63.67 g, and 10.32 g, respectively, which is very close to the predicted value. The result is within the WHO/FAO standard limit. The experimental result shows the mineral composition for Ca, Fe, Zn, and Mg, 169.29 mg, 11.12 mg, 3.84 mg, and 32.48 mg, respectively, which approaches the predicted value. From the mineral content, Zn and Mg are within the WHO recommended limit, but iron and calcium need minor modification to reach the recommended levels. The optimized product's antinutrient composition (phytate, oxalate, and tannin) and *β*-carotene show a close relationship with the predicted value, which is an acceptable limit. Thus, the optimizer gives the predicted response congruous with the actual value recommended for the formulation of infant foods to reach the nutrient requirement.

The results are feasible to develop a nutritious complementary food by compositing oats, chickpea, yellow maize, avocado powder, and Jaggery. The optimal mixtures of 40-49 g/100 g, chickpea: 15-31.77 g/100 g, yellow maize: 12.17-21.6 g/100 g, and avocado powder: 5-11 g/100 g with 10 g/100 g Jaggery enhance significant improvement in the composite food nutritional quality which can contribute to preventing malnutrition in infant food.

## 4. Conclusions

This study was carried out to formulate complementary food to meet the nutritional needs of infants. It was done by mixing readily available raw materials (oat, chickpea, yellow maize, avocado powder, and Jaggery). Thus, this research found that the optimum composite food mixture as the ratio of 40 g of oat, 25.81 g of chickpea, 13.78 g of yellow maize, and 10.41 g of avocado pulp powder with 10 g sugarcane Jaggery is considered a better optimal food composition among the studied components with the response optimizer composite desirability of 0.9085. Also, studied results proved that the appropriate raw materials were processed and formulated well-being complementary food with improved nutritional value suitable for infant feeding and all age hold communities.

## Figures and Tables

**Table 1:** Mixture design for the formulation of complementary foods (CF).

Run order	Oat (g)	Chickpea (g)	Yellow maize (g)	Avocado powder (g)
CF1	40	22	23	5
CF2	41.4	28.8	13.3	6.5
CF3	45	22	12	11
CF4	43.8	23.4	13.3	9.5
CF5	40	27	12	11
CF6	48	25	12	5
CF7	50.1	22.1	12.6	5.2
CF8	53.6	17.3	12.8	6.3
CF9	55	18	12	5
CF10	46.8	23.4	13.3	6.5
CF11	40	33	12	5
CF12	50	15	14	11
CF13	55	15	15	5
CF14	48.6	20.6	12.6	8.2
CF15	51	22	12	5
CF16	52.1	16.6	13.6	7.7
CF17	48.8	20.5	15.1	5.6
Levels:	40-55 g	15-33 g	12-23 g	5-11 g

**Table 2:** Full cubic regression model ANOVA *p* values and model coefficients for proximate composition of formulated complementary food samples.

Source	Moisture	Ash	Protein	Fat	Fiber	CHO	Energy
Linear	≤0.001	≤0.001	≤0.001	≤0.001	0.002	≤0.001	≤0.001
Special cubic	≤0.001	0.001	≤0.001	≤0.001	≤0.001	≤0.001	≤0.001
Oat^∗^chickpea^∗^maize	≤0.001	0.416	≤0.001	≤0.001	0.393	≤0.001	≤0.001
Oat^∗^chickpea^∗^avocado powder	≤0.001	0.692	≤0.001	≤0.001	0.873	≤0.001	≤0.001
Oat^∗^maize^∗^avocado powder	0.046	0.047	0.022	0.048	0.987	0.004	0.264
Chickpea^∗^maize^∗^avocado	≤0.001	0.234	≤0.001	≤0.001	0.829	≤0.001	0.005
*R* ^2^ (adjusted)	0.875	0.864	0.998	0.995	0.976	0.988	0.992

**Table 3:** ANOVA *p* values for full cubic regression model for Ca, Fe, Zn, Mg, Mn, and Cu.

Source	Ca	Fe	Zn	Mg	Mn	Cu
Linear	≤0.001	≤0.001	≤0.001	≤0.001	≤0.001	0.004
Quadratic	≤0.001	≤0.001	≤0.001	≤0.001	0.206	≤0.001
Special cubic	≤0.001	≤0.001	≤0.001	≤0.001	0.096	0.022
Oat^∗^chickpea^∗^maize	≤0.001	≤0.001	≤0.001	≤0.001	0.328	0.125
Oat^∗^chickpea^∗^avocado powder	≤0.001	≤0.001	≤0.001	≤0.001	0.349	0.099
Oat^∗^maize^∗^avocado powder	0.036	≤0.001	≤0.001	0.574	0.329	0.080
Chickpea^∗^maize^∗^avocado powder	≤0.001	≤0.001	≤0.001	≤0.001	0.292	0.086
Full cubic	≤0.001	≤0.001	≤0.001	≤0.001	≤0.001	0.009
*R* ^2^ (adjusted)	0.992	0.992	0.982	0.977	0.330	0.872

**Table 4:** Full cubic regression model ANOVA *p* values and model coefficients for antinutrients, vitamins, and polyphenol of prepared complementary foods.

Source	Phytate (mg/100 g)	Tannin (mg/100 g)	Oxalate (mg/100 g)	*β*-Carotene (mg)	Vit. C (mg)	Polyphenol (mg/100 g)
Linear	≤0.001	0.002	≤0.001	≤0.001	0.453	≤0.001
Quadratic	≤0.001	≤0.001	≤0.001	≤0.001	0.106	≤0.001
Special cubic	≤0.001	≤0.001	≤0.001	≤0.001	0.188	≤0.001
Oat^∗^chickpea^∗^maize	≤0.001	0.002	≤0.001	≤0.001	0.424	0.728
Oat^∗^chickpea^∗^avocado powder	0.008	0.001	≤0.001	≤0.001	0.312	0.841
Oat^∗^maize^∗^avocado powder	≤0.001	0.005	≤0.001	≤0.001	0.058	0.772
Chickpea^∗^maize^∗^avocado powder	≤0.001	0.001	≤0.001	≤0.001	0.173	0.991
Full cubic	≤0.001	0.001	≤0.001	≤0.001	0.030	0.871
*R* ^2^ (adjusted)	0.998	0.890	0.996	0.993	0.893	0.905

**Table 5:** Response optimization using Minitab D-optimizer for formulated food.

Optimized food coding	Composition of food constituents (%)					Overall composite desirability
	Oat	Chickpea	Yellow maize	Avocado powder	Jaggery	
F-1	40	31.2262	12.9589	5.81491	10.00	1.000000
F-2	40	25.8148	13.7778	10.4074	10.00	0.908463
F-3	49.3036	15	14.6964	11	10.00	1.000000
F-4	40.9091	31.5989	12.2789	5.21306	10.00	1.000000

## Data Availability

All the necessary data have been included in the manuscript.
